# Profile and mental health characterization of childhood overprotection/overcontrol experiences among Chinese university students: a nationwide survey

**DOI:** 10.3389/fpsyt.2023.1238254

**Published:** 2023-10-16

**Authors:** Jiamei Zhang, Zhipeng Wu, Haojuan Tao, Min Chen, Miaoyu Yu, Liang Zhou, Meng Sun, Dongsheng Lv, Guangcheng Cui, Qizhong Yi, Hong Tang, Cuixia An, Zhening Liu, Xiaojun Huang, Yicheng Long

**Affiliations:** ^1^Department of Psychiatry, The Second Xiangya Hospital, Central South University, Changsha, Hunan, China; ^2^National Clinical Research Center for Mental Disorders, The Second Xiangya Hospital, Central South University, Changsha, Hunan, China; ^3^Department of Psychiatry, Jining Medical University, Jining, Shandong, China; ^4^Department of Mental Health, The Second Affiliated Hospital of Guangxi Medical University, Nanning, Guangxi, China; ^5^Department of Social Psychiatry, The Affiliated Brain Hospital of Guangzhou Medical University, Guangzhou, Guangdong, China; ^6^Department of Mental Health Institute of Inner Mongolia Autonomous Region, The Affiliated Mental Center of Inner Mongolia Medical University, Hohhot, Inner Mongolia, China; ^7^Department of Psychiatry, Qiqihar Medical University, Qiqihar, Heilongjiang, China; ^8^Xinjiang Clinical Research Center for Mental Disorders, The Psychological Medicine Center, The First Affiliated Hospital of Xinjiang Medical University, Urumqi, Xinjiang, China; ^9^Department of Psychiatry, Gannan Medical University, Ganzhou, Jiangxi, China; ^10^Department of Psychiatry, The First Hospital of Hebei Medical University, Shijiazhuang, Hebei, China; ^11^Department of Psychiatry, Jiangxi Provincial People’s Hospital, The First Affiliated Hospital of Nanchang Medical College, Nanchang, Jiangxi, China

**Keywords:** childhood trauma, overprotection, overcontrol, mental health, depression, psychotic-like experience

## Abstract

**Introduction:**

The childhood experiences of being overprotected and overcontrolled by family members have been suggested to be potentially traumatic. However, the possible associated factors of these experiences among young people are still not well studied. This study aimed to partly fill such gaps by a relatively large, nationwide survey of Chinese university students.

**Methods:**

A total of 5,823 university students across nine different provinces in China were included by the convenience sampling method in the data analyses. All participants completed the overprotection/overcontrol (OP/OC) subscale in a recently developed 33-item childhood trauma questionnaire (CTQ- 33). Data were also collected on all participants’ socio-demographic profiles and characterization of mental health. Binary logistic regression was conducted to investigate the associated socio-demographic and psychological factors of OP/ OC.

**Results:**

The prevalence of childhood OP/OC was estimated as 15.63% (910/5,823) based on a cutoff OP/OC subscale score of  ≥ 13. Binary logistic regression suggested that being male, being a single child, having depression, having psychotic-like experiences, lower family functioning, and lower psychological resilience were independently associated with childhood OP/OC experiences (all corrected-*p* < 0.05). The OP/OC was also positively associated with all the other trauma subtypes (abuses and neglects) in the CTQ-33, while there are both shared and unique associated factors between the OP/OC and other trauma subtypes. Post-hoc analyses suggested that OP/OC experiences were associated with depression in only females and associated with anxiety in only males.

**Discussion:**

Our results may provide initial evidence that childhood OP/OC experiences would have negative effects on young people’s mental health which merits further investigations, especially in clinical populations.

## 1. Introduction

Overprotection/overcontrol (OP/OC) behaviors were defined as behaviors in which caregivers (including parents and other family members) are overly involved in children’s daily activities and experiences, often caused by excessive anxiety about the children’s safety (1, 2). As suggested by past studies, multiple possible reasons may lead to OP/OC behaviors. For example, some parents exhibited fear in fulfilling their parenting responsibilities, which may in turn lead to their OP/OC (3). Furthermore, a lack of care by one parent can also lead to OP/OC behaviors by the other one (4). A prior research study has shown that perceived OP/OC from family members might limit children’s development of a clear understanding of environmental dangers and might have negative effects on their mental health statuses (5, 6). For instance, perceived OP/OC experiences were suggested to be possibly associated with decreased self-efficacy and increased vulnerability to perceived threats (1), the development of childhood anxiety (3), as well as the onset of anorexia (7) in children and teenagers. In addition, OP/OC might be related to increased risks of depression, post-traumatic stress disorder (8), and suicidal behaviors (9).

In addition to the short-term negative psychological effects of OP/OC in children/teenagers as mentioned above, recent studies have also suggested that OP/OC might be developmentally traumatizing, and childhood OP/OC experiences may have long-term effects on one’s mental health in early adulthood and even later life (5, 10, 11). For instance, individual recall of childhood OP/OC appears to be associated with a higher prevalence and incidence of adult psychological health problems in the general population (12–14). Some evidence suggests that childhood OP/OC experiences are related to sleep disturbance (15) and associated with difficult recovery in patients with schizophrenia (16) in adulthood. Another related study reported that overprotective support reduced stress in the short term but hindered individuals from coping with stress in the long run by weakening autonomy, especially when that support is terminated (17). For these reasons, perceived OP/OC during childhood has been regarded as a kind of traumatic experience besides the other well-known childhood trauma subtypes (e.g., abuses and neglects) and attracted attention in recent psychological studies (2, 18). Recognizing and identifying factors associated with childhood OP/OC experiences, therefore, may be valuable for improving our understanding of the developments of common mental problems and disorders, as well as finding potential targets for early interventions for mental disorders.

The current literature about possible associated factors of childhood OP/OC experiences in young people, however, is still limited in several ways. First, some previous results have reported inconsistent and even conflicting conclusions. For example, while many earlier studies as mentioned above suggested that childhood OP/OC is related to more mental problems including depressive and anxiety symptoms in later life, the opposite results were also reported, e.g., that paternal overcontrols predicted lower anxious-depressed symptoms (19). One of the potential reasons for these contradictory results may be the insufficient sample size in many of these studies; for instance, the samples in most of the previous studies range from only dozens to hundreds (20–23), which may lead to relatively low statistical power and unreliable results. Second most of the prior studies have focused on the associations between OP/OC and several common mental problems such as anxiety and depression; however, the knowledge is limited on the relationships between OP/OC and some other important socio-demographic profiles and mental health characteristics. These characteristics include, for example, psychological resilience which is defined as one’s ability to recover and maintain adaptive behaviors when facing constant stress (24). There has been evidence that other subtypes of childhood trauma (e.g., abuses and neglects) could lead to a lower psychological resilience, which mediates the relationships between childhood trauma and depression in college students (25). As a kind of traumatic experiences, OP/OC experiences may be also associated with a lower psychological resilience, which remains however poorly investigated to our knowledge. Third, while most of the prior studies on OP/OC experiences were conducted in Western countries, it is relatively little known about the prevalence and associated factors of OP/OC among youths under other cultural conditions, such as in China. One possible reason for such a limitation is the lack of an easy and feasible screening tool for OP/OC experiences in the Chinese language. Nevertheless, this gap has been addressed by a recently validated Chinese version of the 33-item expanded childhood trauma questionnaire (CTQ-33) (18), and further studies on OP/OC among the young Chinese populations may be warranted.

In the current study, we aim to address the limitations raised above by performing a nationwide, large-sample survey among the young Chinese population. Specifically, a total of 5,823 Chinese university students across nine different provinces in China were included in the analyses. Data were collected on all participants’ childhood OP/OC experiences, socio-demographic profiles, and characterization of mental health (e.g., psychological resilience). Logistic regression models were conducted to investigate the possible associations between childhood OP/OC experiences and other socio-demographic/psychological factors. We hope that our results will shed light on the understanding of the possible role of OP/OC in psychological health among young people.

## 2. Methods

### 2.1. Participants

A total of 5,993 Chinese university students were initially recruited in this survey using the convenience sampling method from nine universities across nine different provinces (Shandong, Jiangxi, Guangxi, Guangdong, Hebei, Inner Mongolia, Heilongjiang, Hunan, and Xinjiang) in China (see distributions in Table 1). The survey was conducted from September 2021 to October 2021, and all students completed the survey online through a famous platform in China, “Questionnaire Star”.^1^ To avoid the potential confounding impacts of other clinical conditions on the results, students with a previous diagnosis of any psychiatric disorder were excluded (*n* = 120). In addition, students with missing data (*n* = 47) or over the age of 25 years (*n* = 3) were excluded. Therefore, 5,823 participants were included in the final data analyses in the current study (see Table 1 for sample characteristics). All participants and/or their guardians gave informed consent to agree to participate in this study. The research proposal was approved by the Ethics Committee of the Second Xiangya Hospital of Central South University.

**Table 1:** Sample characteristics of the analyzed participants.

University	Located province	Number of participants	Females (%)/males	Age (mean ± SD)	Scores of CTQ-OP/OC subscale		
					Mean	SD	Mean + SD
Jining Medical University	Shandong	1,212	694 (57.26)/518	19.56 ± 1.28	8.88	3.64	12.52
Gannan Medical University	Jiangxi	3,065	1717 (56.02)/1,348	19.50 ± 1.56	9.01	3.37	12.38
Guangxi Medical University	Guangxi	317	187 (58.99)/130	19.96 ± 1.31	8.99	3.18	12.17
Guangzhou Medical University	Guangdong	85	51 (60.00)/34	19.49 ± 1.36	9.28	3.14	12.42
Hebei Medical University	Hebei	161	105 (65.22)/56	18.25 ± 0.74	8.61	3.08	11.69
Inner Mongolia Medical University	Inner Mongolia	131	100 (76.34)/31	18.73 ± 0.92	8.15	3.41	11.56
Qiqihar Medical University	Heilongjiang	298	177 (59.40)/121	18.37 ± 0.79	8.62	3.49	12.11
Central South University	Hunan	394	210 (53.30)/184	18.28 ± 1.08	9.08	3.48	12.56
Xinjiang Medical University	Xinjiang	160	102 (63.75)/58	20.01 ± 1.41	8.56	3.57	12.13
Total		5,823	3,343 (57.41)/2,480	19.36 ± 1.47	8.93	3.43	12.36

CTQ, childhood trauma questionnaire; OP/OC, overprotection/overcontrol; SD, standard deviation.

### 2.2. Assessments

#### 2.2.1. Socio-demographic factors

Information on the following socio-demographic factors was collected from all participants and taken into the analyses: age, sex, ethnicity (Han or minority), single-child household (yes or no), parental separation (yes or no), left-behind children experiences (“Are one or both parents have not been with the participants for at least 6 months before the age of 16 years?,” yes or no), as well as family histories of mental disorders. Note that all participants with a personal history of mental disorders have been excluded from the analyses.

#### 2.2.2. Measure of OP/OC experiences

Childhood OP/OC experiences of all participants were measured by the OP/OC subscale of the CTQ-33 (2). The CTQ-33 was expanded from the original 28-item childhood trauma questionnaire (CTQ-28) (26) with an additional OP/OC subscale and thus has six subscales measuring six different subtypes of childhood trauma experiences: emotional abuse, physical abuse, sexual abuse, physical neglect, emotional neglect, and OP/OC (2). All items in the CTQ-33 are 5-point Likert-type questions, and higher scores indicate higher levels of childhood trauma experiences. The Chinese version of the original CTQ-28 has been shown to have good reliability and validity in Chinese populations (27). The additional OP/OC subscale in the CTQ-33 has also been translated into Chinese and proved to be valid (18). In the current study, the CTQ-33 displayed good internal consistency (Cronbach’s α = 0.843).

Based on prior publications, participants with scores above the cutoff points for a particular subscale can be defined as having a particular subtype of childhood trauma experience as follows: physical abuse ≥10, emotional abuse ≥13, sexual abuse ≥8, physical neglect ≥10, and emotional neglect ≥15 (28, 29). In the present study, we intended to first classify all participants into those with and without childhood OP/OC experiences. However, to the best of our knowledge, an optimal cutoff point for the OP/OC subscale in the CTQ-33 has not been established to date. Therefore, referring to multiple published studies (30–32), we estimated the appropriate cutoff score for the OP/OC subscale based on one standard deviation (SD) above the mean score in the surveyed sample. The participants with an OP/OC subscale score higher than such a cutoff point were then defined as having childhood OP/OC experiences.

#### 2.2.3. Self-reported depression

All participants completed the self-reported, 9-item Patient Health Questionnaire (PHQ-9) (33) to assess the severity of depressive symptoms over the past 2 weeks. The Chinese version of PHQ-9 has been validated in a previous study (34). Each item of the PHQ-9 was rated on four values ranging from 0 (“not at all”) to 3 (“nearly every day”). The total score of PHQ-9 can range from 0 to 27, and the participants were regarded to have depression when the total score ≥10 (35). In the present study, the PHQ-9 displayed good internal consistency (Cronbach’s α = 0.903).

#### 2.2.4. Self-reported anxiety

All participants completed the self-reported, 7-item Generalized Anxiety Disorder Scale (GAD-7) (36) to assess their anxiety levels during the last 2 weeks. The Chinese version of GAD-7 has shown good reliability and validity in the Chinese population (37, 38). Each item of the GAD-7 was rated from 0 (“not at all”) to 3 (“nearly every day”). The total score of GAD-7 can range from 0 to 21, and the participants were regarded to have anxiety when the total score ≥10 (37, 38). The GAD-7 displayed good internal consistency in this sample (Cronbach’s α = 0.923).

#### 2.2.5. Psychotic-like experiences

The 15-item version of the Community Assessment of Psychic Experiences (CAPE-15) (39–41) was used to evaluate the psychotic-like experiences of all participants. The Chinese version of the CAPE has been validated and is widely used to assess psychotic-like experiences in Chinese populations (42–45). The CAPE-15 includes 15 items that measured both frequency of and distress associated with a series of common psychotic-like experiences (e.g., subclinical delusions and hallucinations). Both the frequency and distress scores of each item are rated on a four-point Likert scale. Referring to prior studies (46), the participants were regarded to have meaningful psychotic-like experiences when both the mean frequency score and mean distress score were greater than 1.5. The frequency score of each subject showed good internal consistency (Cronbach’s α = 0.871).

#### 2.2.6. Family functioning

The family functioning of each participant was measured by the Family APGAR scale (47, 48). The Chinese version of Family APGAR has been validated and widely used in previous studies (48–50). The Family APGAR scale consists of five items assessing family functioning from five dimensions: adaptation (“A”), partnership (“P”), growth (“G”), affection (“A”), and resolution (“R”). The score of each item ranges from 0 (“almost always”) to 2 (“hardly ever”). Total scores of the Family APGAR scale can thus range from 0 to 10, and a relatively low family functioning can be defined by a total score ≤3 (48–50). The Chinese version of the Family APGAR in our research has good internal consistency (Cronbach’s α = 0.922).

#### 2.2.7. Psychological resilience

Each participant’s psychological resilience was measured by the 10-item Connor-Davidson Resilience Scale (CD-RISC) (51), a self-administered questionnaire extracted from the original 25-item version (52). The Chinese version of CD-RISC has been validated and widely used in previous studies (24, 53, 54). In the CD-RISC, the score of each item ranges from 0 to 4 (0 = “never” to 4 = “almost always”), and the total score ranges from 0 to 40. Referring to prior research (53), the cutoff of a CD-RISC a total score of ≤25 was used to define a relatively low psychological resilience. The Chinese version of the CD-RISC in this sample has good internal consistency (Cronbach’s α = 0.966).

### 2.3. Statistical analyses

Socio-demographic and psychological characteristics were first compared between the participants with and without childhood OP/OC experiences using descriptive statistics. Independent *t*-tests and chi-square tests were used for continuous variables (e.g., age) and categorical variables (e.g., sex), respectively.

In line with some prior studies (55), binary logistic regression analysis was then performed to investigate the possible associations between all socio-demographic/psychological factors (age, sex, years of education, ethnicity, province, single child, parental separation, left-behind experiences, family history of mental disorders, depression, anxiety, psychotic-like experiences, family functioning, and psychological resilience) and childhood OP/OC experiences after adjusting for the confounding effects of other variables. It should be noted that we took all factors into account in regression models and when investigating the relationship between OP/OC and one factor, the possible confounding effects of all the other factors have been excluded. In addition, the province (coded as dummy variables) was controlled in the analyzing models as a variable of no interest. The *p*-values were corrected across the 14 factors using the Benjamini–Hochberg false discovery rate (FDR) corrections, and a corrected *p*-value of < 0.05 was considered to be statistically significant. Moreover, since previous studies have suggested that OP/OC is highly positively correlated with all the other trauma subtypes (abuses and neglects) in the CTQ-33 (2), we did not include other subscales of the CTQ-33 in the regression model to avoid possible multicollinearity problems. Instead, we explored their relationships with OP/OC using separate models in the following supplementary analyses (see later in Section 2.4).

### 2.4. Supplementary analyses

Several supplementary analyses were performed in addition to the main analyses. First, we tested the relationships between OP/OC and other trauma subtypes (abuses and neglects) in the CTQ-33 using Spearman correlations. We also tested whether OP/OC and other trauma subtypes would have similar associated socio-demographic/psychological factors: here, all participants were classified into those with and without a particular subtype of childhood trauma (e.g., psychical abuse, based on the cutoffs mentioned in Section 2.2.2), and separate binary regression models were used to investigate the associated factors of such trauma subtype. Similar to the analyses on OP/OC, the statistical significance was set at an FDR-corrected *p*-value of < 0.05.

Second, considering that sex differences in mental health have been widely reported (56–58), we further explored the possible sex differences in relationships between OP/OC and other factors. Here, similar to analyses in the entire sample, the associated factors of childhood OP/OC experiences were assessed by binary logistic regression models in the male (*N* = 2,480) and female (*N* = 3,343) participants separately, and the statistical significance was still set at an FDR-corrected *p*-value of < 0.05.

### 2.5. Validation analysis

In the current study, we estimated an appropriate cutoff point for the OP/OC subscale at ≥13. To confirm whether the identified associated factors of OP/OC would change when using different cutoff scores, we repeated the regression analyses using two other different cutoff points ≥12 and ≥14, respectively.

## 3. Results

### 3.1. Sample characteristics and estimated cutoff points

Sample characteristics of the analyzed participants are shown in Table 1. The proportion of female participants was 57.41% (3,343/5,823), and the average age was 19.36 years (SD = 1.47) for the entire sample.

Before the analyses, we first compared the OP/OC scores between different provinces to see whether they can be treated as a homogeneous sample. In the entire sample, the (mean + 1SD) value of the OP/OC subscale score was 12.36. Meanwhile, the (mean + 1SD) values of the OP/OC subscale scores were found to be very close across the subsamples from different provinces. Specifically, in most (7/9) of the subsamples, such values were in the range of 12.11–12.56 except in Hebei (11.69) and Inner Mongolia (11.56; Table 1). Nevertheless, these two provinces had relatively small sample size (*N* = 161/131 for Hebei and Inner Mongolia, respectively) which may bias the results. Therefore, we propose that the distributions of OP/OC scores in different provinces were very close, suggesting they can be treated as a homogeneous sample. According to these results, we also propose that an OP/OC subscale score of ≥13 may be an appropriate cutoff to classify those having and not having clinically meaningful OP/OC experiences. Such a cutoff point was also applied in the following analyses.

### 3.2. Group comparisons on socio-demographic and psychological characteristics

Based on the above cutoff point (OP/OC subscale score ≥ 13), the prevalence of OP/OC experiences was estimated as 15.63% (910/5,823) in the current sample. Results of the direct comparisons on all characteristics between the participants with and without OP/OC experiences were shown in Table 2. Compared with those without OP/OC, the participants with OP/OC experiences had a higher proportion of males (*p* < 0.001), a higher proportion of single child (*p* = 0.031), and a higher proportion of “left-behind” children (*p* = 0.006). Compared with those without OP/OC, the participants with OP/OC experiences are more likely to have depression, anxiety, psychotic-like experiences, low family functioning, and low psychological resilience (all *p* < 0.001).

**Table 2:** Comparisons on socio-demographic and psychological characteristics between the participants with and without childhood OP/OC experiences.

Variables	With OP/OC (*n* = 910)	Without OP/OC (*n* = 4,913)	Group comparison
Age (years, mean ± SD)	19.31 ± 1.48	19.37 ± 1.47	*t* = 1.064, *p* = 0.287
Sex	–	–	*χ*^2^ = 115.786, *p* < 0.001^***^
Male, *n* (%)	535 (58.79%)	1945 (39.59%)	–
Female, *n* (%)	375 (41.21%)	2,968 (60.41%)	–
Years of education (mean ± SD)	13.09 ± 1.25	13.11 ± 1.19	*t* = −0.482, *p* = 0.630
Ethnicity	–	–	*χ*^2^ = 0.079, *p* = 0.779
Han, *n* (%)	851 (93.52%)	4,582 (93.26%)	–
Minority, *n* (%)	59 (6.48%)	331 (6.74%)	–
Single child, *n* (%)	300 (32.97%)	1,444 (29.40%)	*χ*^2^ = 4.679, *p* = 0.031^*^
Parental separation, *n* (%)	98 (10.77%)	500 (10.18%)	*χ*^2^ = 0.292, *p* = 0.589
Left-behind experiences, *n* (%)	318 (34.95%)	1,492 (30.37%)	*χ*^2^ = 7.507, *p* = 0.006^**^
FHMD, *n* (%)	17 (1.87%)	78 (1.59%)	*χ*^2^ = 0.376, *p* = 0.540
Depression, *n* (%)	163 (17.91%)	298 (6.07%)	*χ*^2^ = 147.806, *p* < 0.001^***^
Anxiety, *n* (%)	92 (10.11%)	162 (3.30%)	*χ*^2^ = 85.416, *p* < 0.001^***^
Psychotic-like experiences, *n* (%)	355 (39.01%)	710 (14.45%)	*χ*^2^ = 309.885, *p* < 0.001^***^
Low family functioning, *n* (%)	697 (76.59%)	1835 (37.35%)	*χ*^2^ = 481.149, *p* < 0.001^***^
Low psychological resilience, *n* (%)	471 (51.76%)	1,065 (21.68%)	*χ*^2^ = 357.747, *p* < 0.001^***^
Total, *n* (%)	910 (15.63%)	4,913 (84.37%)	–

^***^p < 0.001, ^**^p < 0.01, ^*^p < 0.05. FHMD, family history of mental disorders; OP/OC, overprotection/overcontrol; SD, standard deviation.

### 3.3. Results of binary logistic regression analysis

**Figure 1:**
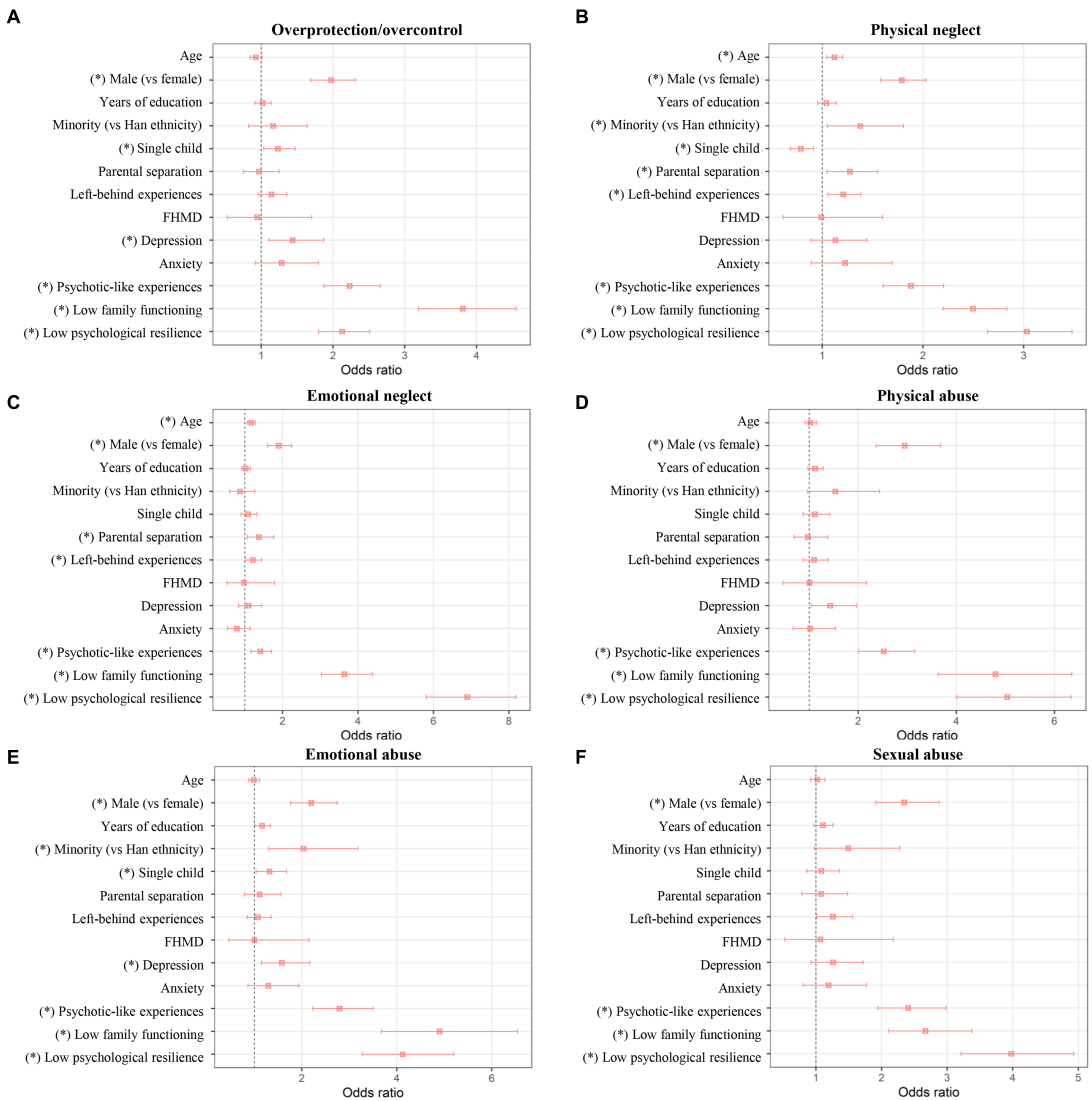
Results of separate binary logistic regression analyses for factors associated with OP/OC **(A)** and for factors associated with other trauma subtypes **(B–F)**. The odds ratios with 95% confidence intervals are presented, and the “*” indicates a significant association with corrected *p* < 0.05. FHMD, family history of mental disorder; OP/OC, overprotection/overcontrol.

As shown in Table 3 and Figure 1A, after controlling for confounding factors in the logistic regression model, the following factors remained independently associated with OP/OC experiences: being male (odds ratio 1.973, 95% confidence interval 1.685–2.311, corrected *p* < 0.001), being a single child (odds ratio 1.232, 95% confidence interval 1.033–1.471, corrected *p* = 0.046), having depression (odds ratio 1.436, 95% confidence interval 1.105–1.866, corrected *p* = 0.018), having psychotic-like experiences (odds ratio 2.231, 95% confidence interval 1.872–2.659, corrected *p* < 0.001), having low family functioning (odds ratio 3.808, 95% confidence interval 3.188–4.549, corrected *p* < 0.001), and having low psychological resilience (odds ratio 2.126, 95% confidence interval 1.799–2.511, corrected *p* < 0.001). There were no statistically significant associations between OP/OC and the following factors: province, age, years of education, ethnicity, parental separation, left-behind experiences, family history of mental disorders, and anxiety (all corrected *p* > 0.05), after controlling for confounding factors.

**Table 3:** Results of the binary logistic regression analysis for factors associated with OP/OC.

Variables	B	SE	Wald	Significance	Odds ratio	95% CI for odds ratio	
						Lower	Upper
Age	−0.080	0.046	3.055	*p* = 0.149	0.923	0.844	1.010
Male (*vs* female)	0.680	0.081	70.915	*p* < 0.001^***^	1.973	1.685	2.311
Years of education	0.017	0.056	0.092	*p* = 0.849	1.017	0.911	1.136
Minority (*vs* Han ethnicity)	0.150	0.175	0.738	*p* = 0.507	1.162	0.825	1.637
Single child	0.209	0.090	5.368	*p* = 0.046^*^	1.232	1.033	1.471
Parental separation	−0.033	0.130	0.065	*p* = 0.849	0.968	0.750	1.248
Left-behind experiences	0.131	0.088	2.215	*p* = 0.218	1.140	0.959	1.354
FHMD	−0.057	0.301	0.036	*p* = 0.849	0.944	0.524	1.702
Depression	0.362	0.134	7.323	*p* = 0.018^*^	1.436	1.105	1.866
Anxiety	0.247	0.172	2.067	*p* = 0.218	1.281	0.914	1.795
Psychotic-like experiences	0.803	0.089	80.433	*p* < 0.001^***^	2.231	1.872	2.659
Low family functioning	1.337	0.091	217.369	*p* < 0.001^***^	3.808	3.188	4.549
Low psychological resilience	0.754	0.085	78.765	*p* < 0.001^***^	2.126	1.799	2.511

The presented *p* values were FDR-corrected. ^***^p < 0.001, ^**^p < 0.01, ^*^p < 0.05. CI, confidence interval; FDR, false discovery rate; FHMD, family history of mental disorder; OP/OC, overprotection/overcontrol; SE, standard error.

### 3.4. Supplementary analyses on other trauma subtypes

As shown in Table 4, significant positive correlations were found between the OP/OC score and scores of all other trauma subtypes in the CTQ-33 (all *p* < 0.001), confirming that OP/OC is highly positively associated with the other trauma subtypes. Results of the separate binary logistic regression analyses for factors associated with other trauma subtypes are shown in Figures 1B–F and Supplementary Tables S1–S5. Generally, it was found that the OP/OC and other trauma subtypes have both shared and unique associated factors (*p* < 0.05 after corrections). For example, all trauma subtypes including OP/OC were found to be positively associated with having psychotic-like experiences, having low family functioning, and having low psychological resilience; meanwhile, parental separation was found to be associated with only the physical neglect and emotional neglect experiences (Figure 1).

**Table 4:** Spearman correlation coefficients between the OP/OC score and scores of other trauma subtypes in the CTQ-33.

	Physical neglect	Emotional neglect	Physical abuse	Emotional abuse	Sexual abuse
OP/OC	0.340^***^	0.380^***^	0.408^***^	0.454^***^	0.353^***^
Physical neglect		0.551^***^	0.392^***^	0.404^***^	0.370^***^
Emotional neglect			0.343^***^	0.338^***^	0.285^***^
Physical abuse				0.559^***^	0.574^***^
Emotional abuse					0.450^***^

CTQ-33, the 33-item childhood trauma questionnaire; OP/OC, overprotection/overcontrol; the “*” indicates a significant correlation with *p* < 0.001.

### 3.5. Supplementary analyses on possible sex differences

**Figure 2:**
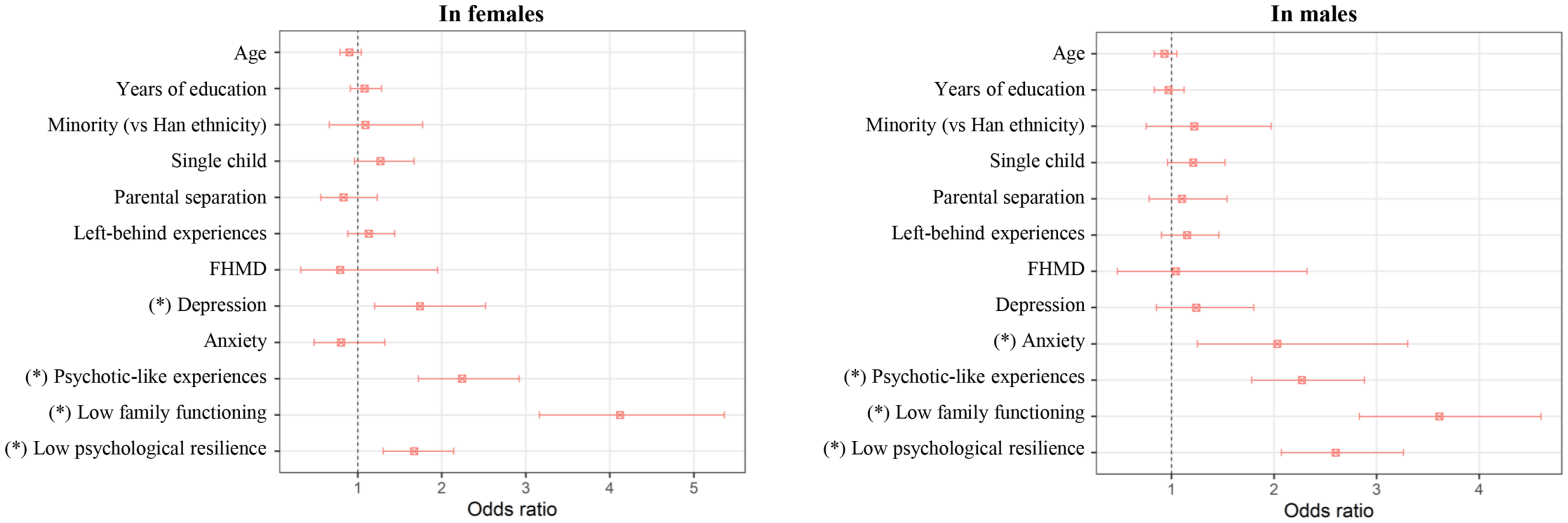
Results of separate binary logistic regression analyses for factors associated with OP/OC in the female or male participants. The odds ratios with 95% confidence intervals are presented, and the “*” indicates a significant association with corrected *p* < 0.05. FHMD, family history of mental disorder; OP/OC, overprotection/overcontrol.

Results of separate logistic regression analyses in the female or male participants are shown in Figure 2 and Supplementary Tables S6, S7. Generally, most of the associated factors of OP/OC were found to be consistent across the female and male participants (corrected *p* < 0.05 in both the two subsamples). The exceptions were that OP/OC was found to be associated with depression in only the female participants, and associated with anxiety in only the male participants (Figure 2).

### 3.6. Validation analysis

When using the cutoff points of OP/OC subscale score ≥12 or ≥14, 20.81% (1,212/5823) and 12.31% (717/5823) of the surveyed participants were categorized as having OP/OC experiences, respectively. The following factors were still found to be significantly associated with OP/OC when using the above different cutoff points: being male, being a single child, having depression, having psychotic-like experiences, having lower family functioning, and having lower psychological resilience (all corrected *p* < 0.05, see Supplementary Tables S8, S9).

## 4. Discussion

In this study, we investigated the possible associations between childhood OP/OC experiences and a series of socio-demographic and psychological factors in a nationwide sample of Chinese university students. Generally, our results suggested multiple non-modifiable (e.g., sex) and modifiable (e.g., family functioning) factors that could be independently associated with childhood OP/OC experiences. The OP/OC was also positively associated with all the other assessed trauma subtypes (abuses and neglects) in the CTQ-33. These results may provide initial evidence that childhood OP/OC experiences might have negative effects on the mental health in young populations.

In the current study, we first explored an appropriate cutoff point for the OP/OC subscale in CTQ-33 based on the statistical distributions in the surveyed sample. The cutoff was estimated at ≥13, and 15.63% (910/5823) of the surveyed participants were categorized as having OP/OC experiences according to such cutoff point. This prevalence is higher than those of physical abuse (8.59%, 500/5823), emotional abuse (8.07%, 470/5823) and sexual abuse (8.98%, 522/5823) but lower than those of physical neglect (33.52%, 1952/5823) and emotional neglect (16.26%, 947/5823) in the current sample. Note that all the identified associated factors of OP/OC were found to be unchanged when using different cutoff points at ≥12 and ≥14 (see Supplementary Tables S8, S9); therefore, the main conclusions in this study are unlikely to be largely driven by different choices in cutoff points.

Using the binary logistic regression model, we found that being male and being a single child are positively associated with childhood OP/OC experiences (Table 3). The observed sex effects on OP/OC are partly consistent with previous research showing sex differences in perceived parenting styles (59). We propose that several biological and social factors might account for such sex differences. For example, boys are favored over girls under the traditional ideology of son preference (60), which may lead to more focus on the boys than girls in some families. For the same reason, the children which are the single child of their family might attract more attention, and even overprotective parental strategies. Notably, we did not find significant associations between parental separation and OP/OC. One possible reason might be that children can be affected differently by whether their parents’ separation was amicable or conflict-ridden (61).

The regression analyses suggested that having depression is independently associated with OP/OC, even after adjusting for possible confounding effects of all other variables (Table 3). To the best of our knowledge, the findings in previously published studies are not totally consistent regarding the possible associations between OP/OC experiences and levels of depressive symptoms in later life. For example, one earlier research reported a strong association between negative parenting behaviors such as overprotection and later depressive symptom (8, 13). However, there is also research suggesting that paternal overcontrols can predict lower depressive symptoms (19). It is noteworthy that compared to most of these studies, our study has a much larger sample size and thus a higher statistical power. Therefore, this study may provide more solid evidence in support of the positive association between OP/OC and depression. In fact, multiple previous studies have also underlined OP/OC and other childhood traumas as predictors of dissociative depression alongside some linkage to the “traumatic narcissism” concept (13, 62), which are in line with our results and give a possible explanation for such relationship.

Our results also suggested that having psychotic-like experiences is independently associated with OP/OC (Table 3). To the best of our knowledge, this study is one of the first reports to suggest a positive relationship between OP/OC and psychotic-like experiences. Psychotic-like experiences are subclinical delusion-like or hallucination-like symptoms, which are related to increased risks of developing subsequent mental disorders (55). Previous studies have shown that some other subtypes of childhood trauma such as abuses and neglects would strongly increase the risks of developing schizophrenia and other psychotic disorders (63, 64), which may be presented as having psychotic-like experiences in the early stage (65). Here, our results suggest that OP/OC, as another subtype of childhood trauma, is also associated with psychotic-like experiences.

Additionally, we found that childhood OP/OC experiences are associated with lower family functioning and lower psychological resilience (Table 3). Both family dysfunction (66) and decreased psychological resilience (67) have been linked to higher risks of developing mental problems. Lower family functioning was also associated with lower wellbeing and higher risks of substance use (68, 69). These findings, together with the observed significant effects of OP/OC on depression and psychotic-like experiences, may thus highlight the unignorable negative effects of OP/OC experiences on young people’s mental health.

As supplementary analyses, we have explored the possible differences in associated factors between OP/OC and other childhood trauma subtypes. It was found that some associated factors, such as having psychotic-like experiences, lower family functioning, and lower psychological resilience, were shared for all different trauma subtypes including OP/OC (Figure 1). Some differences were also found; for example, being a single child was positively associated with OP/OC but negatively associated with physical neglect; furthermore, having depression was positively associated with OP/OC and emotional abuse but not significantly associated with other trauma subtypes (Figure 1). Therefore, while being a subtype of traumatic experiences, there might be both common and unique features between the OP/OC and other trauma subtypes.

We have also explored the possible sex differences in relationships between OP/OC and other factors by performing analyses in the female and male participants separately. Generally, we found that most of the associated factors of OP/OC were consistent across the female and male participants; however, interestingly, the OP/OC experiences were associated with depression in only the female participants and associated with anxiety in only the male participants (Figure 2). There has been ample evidence for significant sex differences in multiple psychological characteristics, e.g., that females are more likely to be affected by depression (58, 70). Here, our results may partly help to further understand the sex differences in these psychological characteristics in the aspect of different influences of childhood OP/OC experiences.

This study has certain limitations. First, because of the nature of the cross-sectional survey, we are unable to establish the causality in relationships between OP/OC experiences and other factors. Therefore, further longitudinal studies are needed to address such a limitation. Second, several self-reported retrospective scales were used in this study, which may lead to memory-related biases. Third, the OP/OC experiences from one’s father, mother, or other family members were not distinguished in the CTQ-33, which might have different associated socio-demographic factors and psychological effects. This limitation may be overcome by using other scales in future studies. Last, while only healthy participants were included in the current study, further studies conducted in clinical populations with mental disorders may provide more important implications for understanding the negative effects of childhood OP/OC experiences.

## 5. Conclusion

In conclusion, this study investigated the possible associated factors of childhood OP/OC experiences in young populations using the CTQ-33 and a relatively large, nationwide sample of Chinese university students. The main findings include that being male, being a single child, having depression, having psychotic-like experiences, having lower family functioning, and having lower psychological resilience were independently associated with childhood OP/OC experiences. The OP/OC was also positively associated with all the other trauma subtypes (abuses and neglects) in the CTQ-33; nevertheless, the OP/OC and other subtypes of trauma were found to have both shared and unique associated factors. Collectively, these results may provide initial evidence that childhood OP/OC experiences would have negative effects on young people’s mental health and highlight the great value of further investigations on OP/OC especially in participants with mental disorders. The results of this survey in healthy Chinese individuals might also provide baseline reference data for potential future studies on OP/OC in clinical populations.

## Data availability statement

The raw data supporting the conclusions of this article will be made available by the authors, without undue reservation.

## Ethics statement

The studies involving humans were approved by Ethics Committee of the Second Xiangya Hospital of Central South University. The studies were conducted in accordance with the local legislation and institutional requirements. The participants provided their written informed consent to participate in this study.

## Author contributions

JZ, ZW, ZL, and YL contributed to the conception and design of the study. JZ, ZW, MC, MY, LZ, MS, DL, GC, QY, HT, CA, ZL, and YL contributed to the data acquisition. JZ, ZW, and YL contributed to the analysis and interpretation of data. JZ and YL drafted the manuscript. HT, MS, and XH revised the manuscript. All authors contributed to the article and approved the submitted version.

## Footnotes

^1^ www.wjx.xn
