# Supplementary material for: Profile and mental health characterization of childhood overprotection/overcontrol experiences among Chinese university students: a nationwide survey
Source: Front Psychiatry. 2023 Oct 16;14:1238254. doi: 10.3389/fpsyt.2023.1238254 (PMC10614290; doi:10.3389/fpsyt.2023.1238254)
Supplement: Supplementary file 1 [file Table_1.DOCX]

**Supplementary Table S1.** Results of the binary logistic regression analysis for factors associated with physical neglect. The presented *p* values were FDR-corrected.

| Variables | B | SE | Wald | Significance | Odds ratio | 95% CI for odds ratio | |
| --- | --- | --- | --- | --- | --- | --- | --- |
|  |  |  |  |  |  | Lower | Upper |
| Age | 0.114 | 0.036 | 9.930 | *p* = 0.002** | 1.120 | 1.044 | 1.203 |
| Male (vs female) | 0.582 | 0.064 | 83.473 | *p* < 0.001*** | 1.789 | 1.579 | 2.027 |
| Years of education | 0.040 | 0.045 | 0.807 | *p* = 0.369 | 1.041 | 0.953 | 1.137 |
| Minority (vs Han ethnicity) | 0.320 | 0.138 | 5.398 | *p* = 0.020* | 1.377 | 1.051 | 1.804 |
| Single child | -0.240 | 0.074 | 10.643 | *p* = 0.001** | 0.787 | 0.681 | 0.909 |
| Parental separation | 0.241 | 0.101 | 5.725 | *p* = 0.017* | 1.273 | 1.045 | 1.551 |
| Left-behind experiences | 0.189 | 0.069 | 7.479 | *p* = 0.006** | 1.208 | 1.055 | 1.382 |
| FHMD | -0.011 | 0.245 | 0.002 | *p* = 0.963 | 0.989 | 0.611 | 1.599 |
| Depression | 0.122 | 0.125 | 0.960 | *p* = 0.327 | 1.130 | 0.885 | 1.442 |
| Anxiety | 0.204 | 0.164 | 1.549 | *p* = 0.213 | 1.227 | 0.889 | 1.692 |
| Psychotic-like experiences | 0.631 | 0.081 | 60.784 | *p* < 0.001*** | 1.880 | 1.604 | 2.204 |
| Low family functioning | 0.914 | 0.065 | 199.249 | *p* < 0.001*** | 2.495 | 2.198 | 2.833 |
| Low psychological resilience | 1.109 | 0.070 | 249.222 | *p* < 0.001*** | 3.031 | 2.641 | 3.478 |

Note: CI, confidence interval; FDR, false discovery rate; FHMD, Family history of mental disorder; SE, standard error; ***, *p* < 0.001; **, *p* < 0.01; *, *p* < 0.05.

**Supplementary Table S2.** Results of the binary logistic regression analysis for factors associated with emotional neglect. The presented *p* values were FDR-corrected.

| Variables | B | SE | Wald | Significance | Odds ratio | 95% CI for odds ratio | |
| --- | --- | --- | --- | --- | --- | --- | --- |
|  |  |  |  |  |  | Lower | Upper |
| Age | 0.153 | 0.047 | 10.601 | *p* = 0.001*** | 1.165 | 1.063 | 1.278 |
| Male (vs female) | 0.639 | 0.085 | 56.662 | *p* < 0.001*** | 1.894 | 1.604 | 2.237 |
| Years of education | 0.019 | 0.058 | 0.102 | *p* = 0.749 | 1.019 | 0.909 | 1.142 |
| Minority (vs Han ethnicity) | -0.140 | 0.190 | 0.539 | *p* = 0.463 | 0.870 | 0.599 | 1.262 |
| Single child | 0.084 | 0.096 | 0.764 | *p* = 0.382 | 1.088 | 0.901 | 1.314 |
| Parental separation | 0.314 | 0.129 | 5.896 | *p* = 0.015* | 1.369 | 1.062 | 1.765 |
| Left-behind experiences | 0.189 | 0.091 | 4.355 | *p* = 0.037** | 1.209 | 1.012 | 1.444 |
| FHMD | -0.026 | 0.310 | 0.007 | *p* = 0.933 | 0.974 | 0.530 | 1.791 |
| Depression | 0.093 | 0.142 | 0.427 | *p* = 0.513 | 1.097 | 0.831 | 1.450 |
| Anxiety | -0.241 | 0.188 | 1.637 | *p* = 0.201 | 0.786 | 0.543 | 1.137 |
| Psychotic-like experiences | 0.343 | 0.098 | 12.180 | *p* < 0.001*** | 1.409 | 1.162 | 1.708 |
| Low family functioning | 1.292 | 0.094 | 187.288 | *p* < 0.001*** | 3.640 | 3.025 | 4.380 |
| Low psychological resilience | 1.931 | 0.087 | 487.612 | *p* < 0.001*** | 6.895 | 5.809 | 8.183 |

Note: CI, confidence interval; FDR, false discovery rate; FHMD, Family history of mental disorder; SE, standard error; ***, *p* < 0.001; **, *p* < 0.01; *, *p* < 0.05.

**Supplementary Table S3.** Results of the binary logistic regression analysis for factors associated with physical abuse. The presented *p* values were FDR-corrected.

| Variables | B | SE | Wald | Significance | Odds ratio | 95% CI for odds ratio | |
| --- | --- | --- | --- | --- | --- | --- | --- |
|  |  |  |  |  |  | Lower | Upper |
| Age | 0.027 | 0.058 | 0.217 | *p* = 0.833 | 1.028 | 0.916 | 1.152 |
| Male (vs female) | 1.081 | 0.113 | 92.022 | *p* < 0.001*** | 2.947 | 2.363 | 3.676 |
| Years of education | 0.113 | 0.071 | 2.577 | *p* = 0.201 | 1.120 | 0.975 | 1.286 |
| Minority (vs Han ethnicity) | 0.427 | 0.236 | 3.281 | *p* = 0.152 | 1.533 | 0.966 | 2.435 |
| Single child | 0.109 | 0.123 | 0.794 | *p* = 0.606 | 1.115 | 0.877 | 1.419 |
| Parental separation | -0.025 | 0.175 | 0.020 | *p* = 0.988 | 0.976 | 0.692 | 1.375 |
| Left-behind experiences | 0.094 | 0.118 | 0.637 | *p* = 0.614 | 1.099 | 0.872 | 1.384 |
| FHMD | 0.006 | 0.391 | 0.000 | *p* = 0.988 | 1.006 | 0.468 | 2.164 |
| Depression | 0.357 | 0.163 | 4.826 | *p* = 0.072 | 1.430 | 1.039 | 1.967 |
| Anxiety | 0.017 | 0.211 | 0.006 | *p* = 0.988 | 1.017 | 0.672 | 1.537 |
| Psychotic-like experiences | 0.924 | 0.115 | 65.034 | *p* < 0.001*** | 2.519 | 2.012 | 3.153 |
| Low family functioning | 1.568 | 0.143 | 120.111 | *p* < 0.001*** | 4.799 | 3.625 | 6.353 |
| Low psychological resilience | 1.617 | 0.117 | 190.773 | *p* < 0.001*** | 5.039 | 4.006 | 6.339 |

Note: CI, confidence interval; FDR, false discovery rate; FHMD, Family history of mental disorder; SE, standard error; ***, *p* < 0.001; **, *p* < 0.01; *, *p* < 0.05.

**Supplementary Table S4.** Results of the binary logistic regression analysis for factors associated with emotional abuse. The presented *p* values were FDR-corrected.

| Variables | B | SE | Wald | Significance | Odds ratio | 95% CI for odds ratio | |
| --- | --- | --- | --- | --- | --- | --- | --- |
|  |  |  |  |  |  | Lower | Upper |
| Age | -0.017 | 0.060 | 0.080 | *p* = 0.848 | 0.983 | 0.875 | 1.105 |
| Male (vs female) | 0.787 | 0.113 | 48.659 | *p* < 0.001*** | 2.197 | 1.761 | 2.740 |
| Years of education | 0.150 | 0.072 | 4.416 | *p* = 0.059 | 1.162 | 1.010 | 1.337 |
| Minority (vs Han ethnicity) | 0.709 | 0.228 | 9.689 | *p* = 0.005** | 2.033 | 1.300 | 3.177 |
| Single child | 0.275 | 0.123 | 4.987 | *p* = 0.048* | 1.317 | 1.034 | 1.677 |
| Parental separation | 0.105 | 0.173 | 0.366 | *p* = 0.681 | 1.110 | 0.791 | 1.559 |
| Left-behind experiences | 0.067 | 0.120 | 0.313 | *p* = 0.681 | 1.069 | 0.846 | 1.352 |
| FHMD | -0.002 | 0.393 | 0.000 | *p* = 0.996 | 0.998 | 0.462 | 2.155 |
| Depression | 0.456 | 0.162 | 7.901 | *p* = 0.011* | 1.578 | 1.148 | 2.169 |
| Anxiety | 0.257 | 0.206 | 1.556 | *p* = 0.306 | 1.293 | 0.864 | 1.935 |
| Psychotic-like experiences | 1.027 | 0.116 | 78.515 | *p* < 0.001*** | 2.792 | 2.225 | 3.503 |
| Low family functioning | 1.589 | 0.147 | 116.129 | *p* < 0.001*** | 4.901 | 3.671 | 6.544 |
| Low psychological resilience | 1.416 | 0.118 | 143.652 | *p* < 0.001*** | 4.123 | 3.270 | 5.197 |

Note: CI, confidence interval; FDR, false discovery rate; FHMD, Family history of mental disorder; SE, standard error; ***, *p* < 0.001; **, *p* < 0.01; *, *p* < 0.05.

**Supplementary Table S5.** Results of the binary logistic regression analysis for factors associated with sexual abuse. The presented *p* values were FDR-corrected.

| Variables | B | SE | Wald | Significance | Odds ratio | 95% CI for odds ratio | |
| --- | --- | --- | --- | --- | --- | --- | --- |
|  |  |  |  |  |  | Lower | Upper |
| Age | -0.017 | 0.060 | 0.080 | *p* = 0.765 | 0.983 | 0.875 | 1.105 |
| Male (vs female) | 0.787 | 0.113 | 48.659 | *p* < 0.001*** | 2.197 | 1.761 | 2.740 |
| Years of education | 0.150 | 0.072 | 4.416 | *p* = 0.237 | 1.162 | 1.010 | 1.337 |
| Minority (vs Han ethnicity) | 0.709 | 0.228 | 9.689 | *p* = 0.139 | 2.033 | 1.300 | 3.177 |
| Single child | 0.275 | 0.123 | 4.987 | *p* = 0.647 | 1.317 | 1.034 | 1.677 |
| Parental separation | 0.105 | 0.173 | 0.366 | *p* = 0.758 | 1.110 | 0.791 | 1.559 |
| Left-behind experiences | 0.067 | 0.120 | 0.313 | *p* = 0.096 | 1.069 | 0.846 | 1.352 |
| FHMD | -0.002 | 0.393 | 0.000 | *p* = 0.857 | 0.998 | 0.462 | 2.155 |
| Depression | 0.456 | 0.162 | 7.901 | *p* = 0.237 | 1.578 | 1.148 | 2.169 |
| Anxiety | 0.257 | 0.206 | 1.556 | *p* = 0.552 | 1.293 | 0.864 | 1.935 |
| Psychotic-like experiences | 1.027 | 0.116 | 78.515 | *p* < 0.001*** | 2.792 | 2.225 | 3.503 |
| Low family functioning | 1.589 | 0.147 | 116.129 | *p* < 0.001*** | 4.901 | 3.671 | 6.544 |
| Low psychological resilience | 1.416 | 0.118 | 143.652 | *p* < 0.001*** | 4.123 | 3.270 | 5.197 |

Note: CI, confidence interval; FDR, false discovery rate; FHMD, Family history of mental disorder; SE, standard error; ***, *p* < 0.001; **, *p* < 0.01; *, *p* < 0.05.

**Supplementary Table S6.** Results of the binary logistic regression analysis for factors associated with overprotection/overcontrol experiences in the female participants. The presented *p* values were FDR-corrected.

| Variables | B | SE | Wald | Significance | Odds ratio | 95% CI for odds ratio | |
| --- | --- | --- | --- | --- | --- | --- | --- |
|  |  |  |  |  |  | Lower | Upper |
| Age | -0.101 | 0.069 | 2.105 | *p* = 0.294 | 0.904 | 0.789 | 1.036 |
| Years of education | 0.073 | 0.087 | 0.707 | *p* = 0.481 | 1.076 | 0.907 | 1.275 |
| Minority (vs Han ethnicity) | 0.081 | 0.251 | 0.106 | *p* = 0.745 | 1.085 | 0.664 | 1.774 |
| Single child | 0.235 | 0.142 | 2.734 | *p* = 0.235 | 1.265 | 0.957 | 1.672 |
| Parental separation | -0.182 | 0.199 | 0.836 | *p* = 0.481 | 0.834 | 0.564 | 1.231 |
| Left-behind experiences | 0.119 | 0.126 | 0.891 | *p* = 0.481 | 1.127 | 0.879 | 1.443 |
| FHMD | -0.241 | 0.463 | 0.271 | *p* = 0.658 | 0.786 | 0.317 | 1.948 |
| Depression | 0.551 | 0.191 | 8.370 | *p* = 0.012* | 1.736 | 1.195 | 2.522 |
| Anxiety | -0.222 | 0.256 | 0.750 | *p* = 0.481 | 0.801 | 0.484 | 1.324 |
| Psychotic-like experiences | 0.806 | 0.135 | 35.763 | *p* < 0.001*** | 2.239 | 1.719 | 2.916 |
| Low family functioning | 1.415 | 0.135 | 110.252 | *p* < 0.001*** | 4.118 | 3.162 | 5.363 |
| Low psychological resilience | 0.510 | 0.127 | 16.194 | *p* < 0.001*** | 1.665 | 1.299 | 2.135 |

Note: CI, confidence interval; FDR, false discovery rate; FHMD, Family history of mental disorder; SE, standard error; ***, *p* < 0.001; **, *p* < 0.01; *, *p* < 0.05.

**Supplementary Table S7.** Results of the binary logistic regression analysis for factors associated with overprotection/overcontrol experiences in the male participants. The presented *p* values were FDR-corrected.

| Variables | B | SE | Wald | Significance | Odds ratio | 95% CI for odds ratio | |
| --- | --- | --- | --- | --- | --- | --- | --- |
|  |  |  |  |  |  | Lower | Upper |
| Age | -0.068 | 0.062 | 1.214 | *p* = 0.271 | 0.934 | 0.828 | 1.054 |
| Years of education | -0.034 | 0.075 | 0.205 | *p* = 0.651 | 0.967 | 0.834 | 1.120 |
| Minority (vs Han ethnicity) | 0.197 | 0.246 | 0.641 | *p* = 0.423 | 1.218 | 0.751 | 1.974 |
| Single child | 0.187 | 0.118 | 2.494 | *p* = 0.114 | 1.205 | 0.956 | 1.519 |
| Parental separation | 0.092 | 0.175 | 0.278 | *p* = 0.598 | 1.097 | 0.778 | 1.544 |
| Left-behind experiences | 0.138 | 0.124 | 1.230 | *p* = 0.267 | 1.148 | 0.900 | 1.464 |
| FHMD | 0.040 | 0.410 | 0.009 | *p* = 0.923 | 1.040 | 0.466 | 2.324 |
| Depression | 0.211 | 0.191 | 1.216 | *p* = 0.270* | 1.235 | 0.849 | 1.796 |
| Anxiety | 0.709 | 0.248 | 8.192 | *p* = 0.004** | 2.031 | 1.250 | 3.301 |
| Psychotic-like experiences | 0.818 | 0.122 | 44.970 | *p* < 0.001*** | 2.266 | 1.784 | 2.878 |
| Low family functioning | 1.283 | 0.124 | 107.277 | *p* < 0.001*** | 3.607 | 2.830 | 4.599 |
| Low psychological resilience | 0.956 | 0.116 | 67.933 | *p* < 0.001*** | 2.601 | 2.072 | 3.264 |

Note: CI, confidence interval; FDR, false discovery rate; FHMD, Family history of mental disorder; SE, standard error; ***, *p* < 0.001; **, *p* < 0.01; *, *p* < 0.05.

**Supplementary Table S8.** Results of the binary logistic regression analysis for factors associated with OP/OC when using a different cutoff point at OP/OC subscale score <= 12. The presented *p* values were FDR-corrected.

| Variables | B | SE | Wald | Significance | Odds ratio | 95% CI for odds ratio | |
| --- | --- | --- | --- | --- | --- | --- | --- |
|  |  |  |  |  |  | Lower | Upper |
| Age | -0.104 | 0.041 | 6.350 | *p* = 0.022* | 0.901 | 0.831 | 0.977 |
| Male (vs female) | 0.635 | 0.072 | 77.457 | *p* < 0.001*** | 1.888 | 1.639 | 2.175 |
| Years of education | 0.050 | 0.051 | 0.974 | *p* = 0.383 | 1.052 | 0.952 | 1.162 |
| Minority (vs Han ethnicity) | 0.166 | 0.156 | 1.128 | *p* = 0.383 | 1.180 | 0.869 | 1.602 |
| Single child | 0.219 | 0.081 | 7.225 | *p* = 0.015* | 1.244 | 1.061 | 1.459 |
| Parental separation | -0.006 | 0.116 | 0.003 | *p* = 0.956 | 0.994 | 0.792 | 1.247 |
| Left-behind experiences | 0.288 | 0.079 | 13.375 | *p* < 0.001*** | 1.333 | 1.143 | 1.555 |
| FHMD | -0.084 | 0.275 | 0.094 | *p* = 0.822 | 0.919 | 0.536 | 1.576 |
| Depression | 0.286 | 0.127 | 5.046 | *p* = 0.041* | 1.331 | 1.037 | 1.707 |
| Anxiety | 0.167 | 0.165 | 1.028 | *p* = 0.383 | 1.182 | 0.856 | 1.633 |
| Psychotic-like experiences | 0.894 | 0.083 | 116.146 | *p* < 0.001*** | 2.444 | 2.078 | 2.876 |
| Low family functioning | 1.143 | 0.077 | 217.667 | *p* < 0.001*** | 3.137 | 2.695 | 3.651 |
| Low psychological resilience | 0.731 | 0.078 | 88.847 | *p* < 0.001*** | 2.077 | 1.784 | 2.418 |

Note: CI, confidence interval; FDR, false discovery rate; FHMD, Family history of mental disorder; OP/OC, overprotection/overcontrol; SE, standard error; ***, *p* < 0.001; **, *p* < 0.01; *, *p* < 0.05.

**Supplementary Table S9.** Results of the binary logistic regression analysis for factors associated with OP/OC when using a different cutoff point at OP/OC subscale score <= 14. The presented *p* values were FDR-corrected.

| Variables | B | SE | Wald | Significance | Odds ratio | 95% CI for odds ratio | |
| --- | --- | --- | --- | --- | --- | --- | --- |
|  |  |  |  |  |  | Lower | Upper |
| Age | -0.067 | 0.050 | 1.792 | *p* = 0.335 | 0.935 | 0.848 | 1.032 |
| Male (vs female) | 0.760 | 0.090 | 70.880 | *p* < 0.001*** | 2.138 | 1.791 | 2.551 |
| Years of education | 0.030 | 0.061 | 0.232 | *p* = 0.683 | 1.030 | 0.913 | 1.162 |
| Minority (vs Han ethnicity) | 0.206 | 0.190 | 1.174 | *p* = 0.363 | 1.229 | 0.847 | 1.784 |
| Single child | 0.255 | 0.099 | 6.602 | *p* = 0.026* | 1.291 | 1.062 | 1.568 |
| Parental separation | -0.188 | 0.149 | 1.599 | *p* = 0.335 | 0.829 | 0.619 | 1.109 |
| Left-behind experiences | 0.112 | 0.098 | 1.315 | *p* = 0.363 | 1.118 | 0.924 | 1.354 |
| FHMD | 0.071 | 0.319 | 0.049 | *p* = 0.825 | 1.073 | 0.574 | 2.006 |
| Depression | 0.333 | 0.143 | 5.421 | *p* = 0.043* | 1.394 | 1.054 | 1.845 |
| Anxiety | 0.133 | 0.184 | 0.522 | *p* = 0.556 | 1.142 | 0.796 | 1.639 |
| Psychotic-like experiences | 0.854 | 0.097 | 78.019 | *p* < 0.001*** | 2.349 | 1.943 | 2.839 |
| Low family functioning | 1.513 | 0.107 | 201.116 | *p* < 0.001*** | 4.541 | 3.684 | 5.598 |
| Low psychological resilience | 0.902 | 0.093 | 93.526 | *p* < 0.001*** | 2.465 | 2.053 | 2.959 |

Note: CI, confidence interval; FDR, false discovery rate; FHMD, Family history of mental disorder; OP/OC, overprotection/overcontrol; SE, standard error; ***, *p* < 0.001; **, *p* < 0.01; *, *p* < 0.05.
